# Rabies Vaccination Targets for Stray Dog Populations

**DOI:** 10.3389/fvets.2017.00052

**Published:** 2017-04-13

**Authors:** Tiffany Leung, Stephen A. Davis

**Affiliations:** ^1^School of Science, RMIT University, Melbourne, VIC, Australia; ^2^School of Mathematics and Statistics, University of Melbourne, Parkville, VIC, Australia

**Keywords:** dog rabies, canine rabies, mathematical model, infectious disease modeling, zoonosis

## Abstract

The role of stray dogs in the persistence of domestic dog rabies, and whether removal of such dogs is beneficial, remains contentious issues for control programs seeking to eliminate rabies. While a community might reach the WHO vaccination target of 70% for dogs that can be handled, the stray or neighborhood dogs that are too wary of humans to be held are a more problematic population to vaccinate. Here, we present a method to estimate vaccination targets for stray dogs when the dog population is made up of stray, free-roaming, and confined dogs, where the latter two types are considered to have an identifiable owner. The control effort required for stray dogs is determined by the *type-reproduction number, T*_1_, the number of stray dogs infected by one rabid stray dog either directly or via *any* chain of infection involving owned dogs. Like the basic reproduction number *R*_0_ for single host populations, *T*_1_ determines the vaccination effort required to control the spread of disease when control is targeted at one host type, and there is a mix of host types. The application of *T*_1_ to rabies in mixed populations of stray and owned dogs is novel. We show that the outcome is sensitive to the vaccination coverage in the owned dog population, such that if vaccination rates of owned dogs were too low then no control effort targeting stray dogs is able to control or eliminate rabies. The required vaccination level also depends on the composition of the dog population, where a high proportion of either stray or free-roaming dogs implies unrealistically high vaccination levels are required to prevent rabies. We find that the required control effort is less sensitive to continuous culling that increases the death rate of stray dogs than to changes in the carrying capacity of the stray dog population.

## 1 Introduction

Rabies is a preventable infectious disease in warm-blooded animals that causes acute encephalitis and death. The etiological agent is a virus belonging to the genus *Lyssavirus*. Canine rabies is the form carried by domestic dogs that is overwhelmingly responsible for approximately 59,000 human deaths per year (1) where transmission of the virus occurs via a dog bite.

Despite the presence of rabies control programs, rabies remains endemic in over 80 countries (2). The main component of these programs is vaccination, where the long-held World Health Organization (WHO) recommendation of a dog vaccination target of 70% (2, 3) is most often the aim. What we now increasingly appreciate is that differences in the local ecology of the dog population (4), and the dogs’ relationships with the humans they live with (5), can determine the outcome of a control program (6, 7).

The efficacy of a vaccination program is expected to depend on vaccinated dogs living long enough for herd immunity to build up. A relatively rapid turnover in a dog population (where a population is largely made up of stray dogs, for example) ought to decrease the efficacy of a vaccination program (8, 9). Field studies that have measured turnover rates found they are higher in regions with higher dog density, and they are especially high in areas where the stray dog population is high (10). Such thinking and observations have led to the Animal Birth Control program implemented in India, for example, to sterilize the female dogs that were captured and vaccinated, conscious that this would improve the survival of these dogs (no costs of reproduction), reduce population turnover, and increase the impact of the vaccination effort (11, 12).

The proportion of the dog population that is stray (ownerless) varies considerably from one country to another, ranging from 5% in Tanzania (13), 19% in Sri Lanka (6), to as high as 60% in India (12). Stray dogs are typically wary of humans such that they cannot be held, and so vaccination of these dogs needs to be achieved either by physically capturing the dogs or by distributing oral baits (2, 14). In either case, reaching a sufficiently high vaccination coverage of the *whole* dog population becomes a more difficult and costly endeavor.

Another contributing factor to the success of vaccination programs is the relationships that dogs share with the humans they live with. Some owned dogs are fully confined, and others partially free-roaming (6). The degree of care humans provide for dogs ranges from none at all, to supplying food and/or shelter only (but not claiming ownership), to full adoption of the dog as a pet where the dog would be provided food, shelter, and health care (5). By contrast, some claim ownership but provide no care at all (15). These differences can have a considerable effect on the dog’s health and exposure to diseases.

A widely used epidemiological measurement of infectious disease transmission is the basic reproduction number, or *R*_0_, which describes the average number of secondary cases produced by a typical infectious individual in a completely susceptible population (16). It can be interpreted as the initial growth rate of the spread of an infectious disease. It follows that when *R*_0_ > 1, the disease will cause an epidemic in the population, and when *R*_0_ < 1, the disease will die out. Theoretical literature shows how *R*_0_ is calculated and then applied to public health, such that a vaccination coverage of 1 − 1/*R*_0_ is expected to lead to the elimination of a disease (16, 17). However, this assumes that coverage occurs homogeneously across the population. In the context of dog rabies, this may not be the case since dog rabies vaccination campaigns typically target specific types of dogs, such as strays (11, 18). When control targets a single type of host then the required effort can be measured by the type-reproduction number (19).

The type-reproduction number, *T*_1_, is an epidemiological quantity introduced by Roberts and Heesterbeek (19) that measures the effort required to prevent outbreaks when control targets a single type of host. For homogeneous populations, its threshold coincides with *R*_0_. In a heterogeneous population, say, with three host types, the concept of *T*_1_ supposes there is a single infectious type 1 host in an otherwise fully susceptible population; *T*_1_ is the average number of type 1 infections caused by the primary case, either directly or via *any* chain of infection involving host types 2 and 3 (each chain starts with the initial infected type 1 host and ends in a second type 1 infection). Similar to *R*_0_, the critical fraction of host type 1 vaccination coverage required to prevent outbreaks in the entire population is 1 − 1/*T*_1_. We use this relationship to present results on vaccination targets for stray dogs.

The key to interpreting *T*_1_ and how it relates to vaccination coverage targets is that there are three cases to consider. The first is that *T*_1_ > 1. This implies that *R*_0_ > 1 (19) and that control efforts that target type 1 have the potential to bring an outbreak under control, or prevent outbreaks from occurring, provided a vaccination coverage of 1 − 1/*T*_1_ can be achieved. The second is where *T*_1_ < 1 which implies that *R*_0_ < 1 (20) so no control efforts need be applied to type 1 (or indeed to any type). The third and final case is where transmission between the other host types (types 2 and 3, for example) occurs enough such that even if *every* type 1 host were vaccinated or removed, outbreaks would still occur among the other types. Mathematically, this coincides with a single infected stray dog causing an infinite number of stray dog infections because the chains of infection in the owned dog host types never stop.

In this paper, we consider the control of canine rabies in a mixed dog population consisting of strays, owned free-roaming dogs, and owned confined dogs. In particular, we show how *T*_1_ can be calculated from a differential equation model for rabies transmission, and how it is related to *R*_0_. Our goal is to understand how vaccination targets for strays, determined by the quantity *T*_1_, are affected by the composition of the dog population, the numbers of dogs (dog density), the vaccination coverage of the owned dog population, and the mortality rates of the stray population.

The mathematics we use is presented in discrete boxes and contained as figures. The reader will be able to “skip” the mathematics if they wish to do so and still follow the methods, results, and conclusions of the paper, provided they understand the epidemiological quantities, *T*_1_ and *R*_0_.

## 2 Materials and Methods

Motivated by the studies of dog populations by Matter et al. (6) and Massei et al. (5), who both observed differences in dog ownership styles, our modeling approach divides the dog population into three ownership types: stray (type 1), owned free-roaming (type 2), and owned confined (type 3). In our mathematical model of dog rabies transmission, we define stray dogs to be ownerless, hence have no health care, and must forage for their own food. Owned free-roaming dogs are defined to be regularly fed by the community but are provided little to no health care. Owned confined dogs are regularly fed and have access to health care through their owners. Under these differences, stray, owned free-roaming, and owned confined dogs have short, medium, and long life spans, respectively.

A difference in life span between groups of dogs creates a slight epidemiological difference (12, 21) because high natural mortality or additional density-dependent mortality implies that an exposed stray could die of natural means before becoming rabid, whereas this is much more unlikely for an owned dog. The stronger epidemiological differences between the host types are in terms of exposure, and therefore biting rates and transmission to other dogs. Our premise is that an owned confined dog, unless it is momentarily free to roam during hunting or some other activity, will not normally be free to come across or attack other dogs, and hence, is assumed not to wander beyond their owner’s property.

### 2.1 SEI Canine Rabies Model with Three Dog Ownership Types

**Figure 1:**
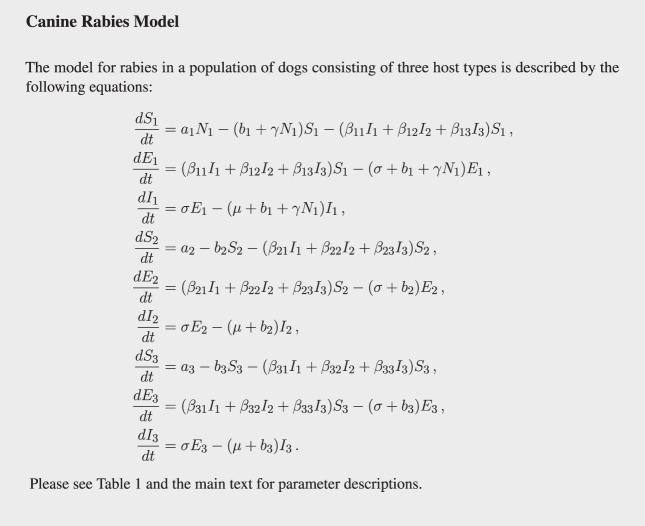
**System of equations for the model of canine rabies with three dog ownership types**.

**Figure 2:**
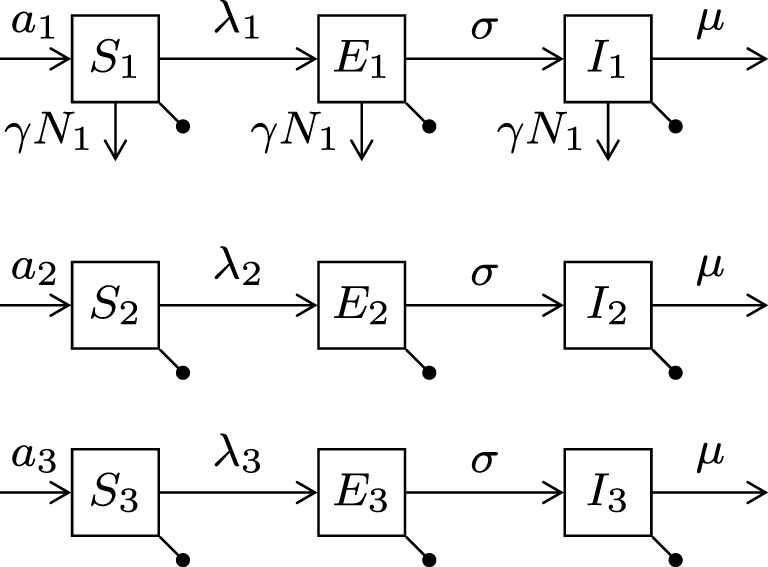
**Diagram of the canine rabies model with three dog ownership types**. Type *i* = (1, 2, 3) represent stray dogs; owned free-roaming dogs; and owned confined dogs, respectively. The bullets represent the natural death rates *b_i_*. Infection of susceptible dogs occurs at a rate *λ_i_* = *β_i_*_1_*I*_1_ + *β_i_*_2_*I*_2_ + *β_i_*_3_*I*_3_, also known as the force of infection. Stray dogs (type 1) face an additional density-dependent mortality at rate *γN*_1_. See Table 1 for parameter meanings.

We extend the susceptible-exposed-infectious (SEI) compartment model typically adopted for rabies (22–24) by sub-dividing the dog population into the three types and hence having nine equations (see Figure 1). Birth and death rates of type *i* are *a_i_* and *b_i_*, respectively (for type *i* = 1, 2, 3). Stray dogs face an additional per capita density-dependent death rate *γN*_1_ (proportional to the stray dog population size). Susceptible (S) dogs become infected at a rate proportional to the number of infectious dogs of each type. Before becoming infectious (I), they enter the exposed (E) state, where they are infected but not yet infectious, for an average period of 1/*σ*. Disease always ends in death at disease mortality rate μ. The flow diagram of the model is presented in Figure 2.

The transmission rates within and between the three types of dogs are given by the transmission matrix *β*, where the element *β_ij_* represents the transmission rate to type *i* from type *j*, for *i, j* = (1, 2, 3). Throughout the paper, we impose that owned free-roaming dogs and stray dogs both roam freely all of the time, so that they are at the highest risk of infection in terms of exposure. Confined dogs are at the lowest risk of infection as they spend minimal time outside their owner’s property. Mathematically, the transmission rates between the three types of dogs (*β_ij_*) are now determined by whether or not the dogs are confined. This simplifies the transmission matrix to contain three different rates: *p*, between two unconfined dogs; *q*, between one confined dog and one unconfined dog; and *r*, between two confined dogs.

Parameter values for the average life expectancy of a stray dog (1/*b*_1_ = 3 years), average exposed (1/*σ* = 25.5 days), and infectious periods (1/μ = 5.7 days) were taken from Hampson et al. (23). The average life expectancy of the confined dog (1/*b*_3_) is set at 8 years, reasonably within the range Patronek et al. (25) determined for owned pet dogs. Owned free-roaming dogs live for 5 years on average (1/*b*_2_), a value chosen to be between that of the average confined and stray dogs. Elements of the transmission matrix *β* remain fixed throughout the paper, at values chosen ((*p, q, r*) = (0.4, 0.04, 0.004) × 10^−3^ per dog per day) so that for a population size of 1,000 composed of 50% stray, 25% owned free-roaming, and 25% owned confined (these proportions are arbitrary), the model has an *R*_0_ = 1.62, a value in line with other studies of dog rabies (8, 23, 26). We chose *p* > *q* > *r* to reflect that an infectious owned confined dog is more likely to be restrained should the owner observe their dog exhibit uncharacteristic aggression. See Table 1 for baseline values of the model.

**Table 1:** **Parameter meanings and baseline values of the SEI canine rabies model with three dog ownership types**.

Parameter	Biological meaning	Baseline value	Reference
*a*_1_	Per capita birth rate of stray dogs	0.0027/day	Assumption
*a*_2_	Birth rate of owned free-roaming dogs	(variable) dogs/day	Assumption
*a*_3_	Birth rate of owned confined dogs	(variable) dogs/day	Assumption
1/*b*_1_	Average natural life span of stray dogs	3 years	(23)
1/*b*_2_	Average natural life span of owned free-roaming dogs	5 years	Assumption
1/*b*_3_	Average natural life span of owned confined dogs	8 years	(25)
1/*σ*	Average incubation period	25.5 days	(23)
1/μ	Average infectious period	5.7 days	(23)
*γ*	Strength of density-dependent mortality	(variable)/(dogs × day)	
*β*	*β_ij_*: transmission term that a dog of type *j* infects a dog of type *i* (*i, j* = 1, 2, 3)	$\left(\begin{array}{ccc} 0.4 & 0.4 & 0.04\\ 0.4 & 0.4 & 0.04\\ 0.04 & 0.04 & 0.004 \end{array}\right)\times 10^{-3}/\left(\text{dog}\times \text{day}\right)$	Assumption

*Parameters with values that vary throughout the paper are marked as (variable). See Figure 5 for details on the form of the transmission matrix*.

Numerical integration of the system was performed using MATLAB (27) with an integration time of 125 years to allow the system to settle to its endemic equilibrium, which is the solution to the differential equations (where the numbers of dogs no longer change over time) in the presence of disease.

### 2.2 Calculation of *R*_0_ and *T*_1_ of the Model

To estimate *R*_0_ for the model, and hence the control effort required to eliminate the virus, we define a next-generation matrix, *K* (28). This methodology has been used for numerous human and wildlife disease systems such as Lyme disease (29). The matrix can be found via a system of differential equations (i.e., those in Figure 1) or by direct epidemiological reasoning (28). Such a matrix consists of elements *k_ij_*, that enumerate, on average, how many infected of one type (type *i*) a single infected of another type (type *j*) would produce over its entire infectious lifetime (28). For example, the average number of confined dogs (type 3) infected by a single rabid stray dog (type 1) is given by *k*_31_.

**Figure 3:**
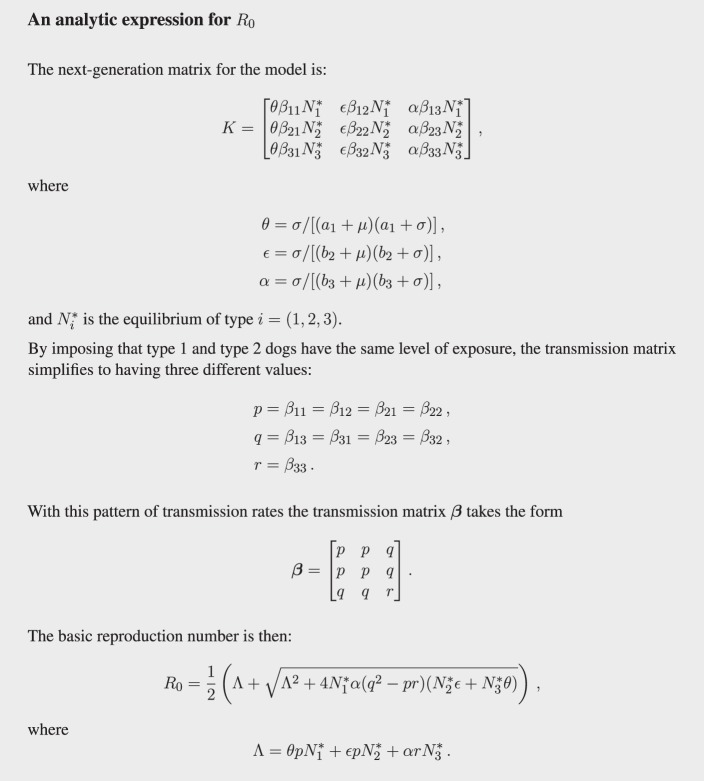
**Analytic expression of *R*_0_ using the next-generation matrix**.

**Figure 4:**
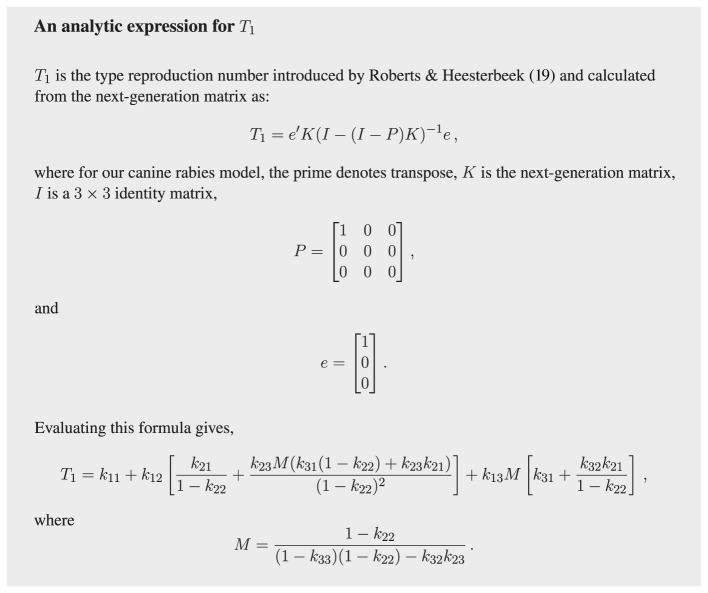
**Analytic expression for the type-reproduction number in terms of the elements (the *k_ij_*) of the next-generation matrix *K***.

In creating a next-generation matrix, it is critical that type is not an attribute that a host can change over time. This means we must make the simplifying assumption that dogs do not change from one type to another over their lifetime, acknowledging this surely does occur to some degree. The simplification makes possible the explicit calculation of *R*_0_ (see Figure 3) and, more importantly, the calculation of vaccination targets for the stray dog population (see Figure 4) without resorting to numerically solving large systems of differential equations.

Values of *R*_0_ and *T*_1_ were calculated for different compositions of dog types and different population sizes. The sizes were chosen to indicate the effects of higher density and therefore higher transmission (because the model assumes that contacts are density-dependent). Initial type population sizes (*N*_1_, *N*_2_, and *N*_3_) were systematically assigned values from 0 to *N* by steps of 25, for *N* = 1,000, 2,000, and 5,000 dogs, where *N* = *N*_1_ + *N*_2_ + *N*_3_. This allows the composition of each dog type to range from 0 to 100%, and all combinations of dog type composition are obtained. Birth rates of owned dogs (*a*_2_ and *a*_3_) and the strength of density-dependent mortality (*γ*) for stray dogs were determined by the assigned initial population size of each type and analytically derived by the expressions: *N*_1_ = *S*_1_ = (*a*_1_ − *b*_1_)/*γ, N*_2_ = *S*_2_ = *a*_2_/*b*_2_, *N*_3_ = *S*_3_ = *a*_3_/*b*_3_. Indeed, this is the disease-free equilibrium, which is the solution to the differential equations (where the numbers of dogs no longer change over time) in the absence of disease. For example, for initial population size *N* = 1,000 and *N*_3_ = 250, we can use the relationship *N*_3_ = *a*_3_/*b*_3_ (as the natural death rate *b_i_* is fixed) to solve for the birth rate of owned confined dogs (*a*_3_ = *N*_3_ × *b*_3_ ≈ 0.0856).

## 3 Results

**Figure 5:**
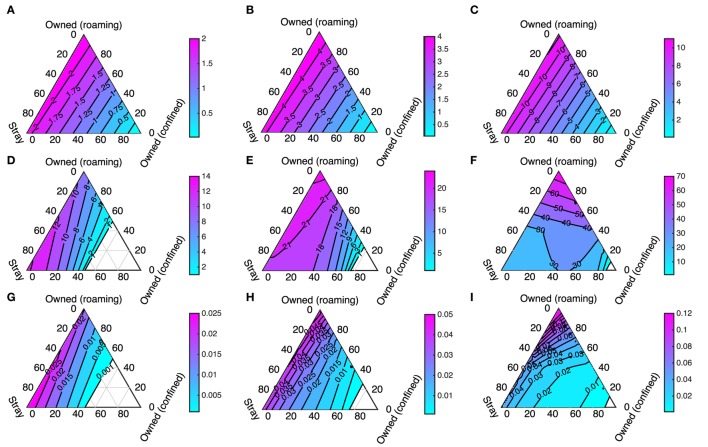
***R*_0_ (first row: (A–C)), number of infected dogs at equilibrium (second row: (D–F)), and rabies prevalence (third row: (G–I)) calculated for three different initial total population size: 1,000 (first column: (A,D,G)); 2,000 (second column: (B,E,H)); and 5,000 (third column: (C,F,I))**. The corners of each triangle represent when populations contain only dogs of one type (as labeled). The edges represent when populations contain two dog types. The center of the triangle represents when the three dog types each make up an equal percentage of the population (totaling 100%).

We first visualize the results of solving the differential equation model given in Figure 1 while varying the initial composition of the dog population and the initial size of the dog population. The initial proportions of dogs that are stray, free-roaming, and confined are varied but always sum to 1. This means that the effects of the composition of the dog population on *R*_0_; the number of infected dogs at equilibrium; and rabies prevalence can all be visualized as ternary plots (see Figure 5). The corners of each of the pictured triangles represent dog populations at the extremes where there are only strays, or only owned free-roaming dogs, or only owned confined dogs. The sides of the triangle represent when the dog population is a mix of two dog types, and the region inside the triangle represents populations that are a mix of all three.

### 3.1 *R*_0_ and Rabies Prevalence

The first row of panels in Figures 5A–C is ternary plots of *R*_0_ and shows that the highest values occur when the dog population consists entirely of owned free-roaming dogs (top corners of the triangles). Slightly lower values of *R*_0_ occur when there is a mix of stray and owned free-roaming dogs, and *R*_0_ drops rapidly when an increasing part of the dog population is owned and confined. These patterns are unaffected by higher initial population sizes; higher dog densities uniformly increase *R*_0_. The peak *R*_0_ value occurs when the entire population is owned and free-roaming (rather than when all dogs are stray) because owned free-roaming dogs have higher survival and hence an exposed dog is less likely to die of natural causes before becoming rabid.

The second row of panels in Figures 5D–F is ternary plots of the total number of infected dogs when the differential equation model settles to an equilibrium. In this case, there are interesting differences between the plots for different initial dog populations. At low densities the worst case scenario (highest numbers of infected dogs) occurs when the dog population consists mostly of stray dogs, whereas at higher densities higher numbers of infected dogs arise when the population consists mostly of owned free-roaming dogs. The contour line where there is one infected dog in the total population (Figures 5D–F) is roughly equivalent to when *R*_0_ = 1 (Figures 5A–C). Where there is an “empty” triangle, the total number of infected dogs (in the “exposed” and “infectious” state) is less than one, which coincides with when *R*_0_ < 1. This “empty” triangle becomes smaller for higher initial dog densities. There is a highly non-linear contour line at 30 infected dogs in Figure 5F. The differences in birth rates and life spans between the host types cause both the non-linear contour line and the shift from maximum numbers of infected dogs occurring when all dogs are stray (at *N* = 1,000) to all dogs are owned free-roaming (at *N* = 2,000 and *N* = 5,000). For the stray dogs, the lower population sizes are associated with higher density-dependent mortality and hence greater population turnover, while for the owned dogs their life spans are fixed, and only the birth rate varies when population sizes are lower or higher.

The final row of panels in Figures 5G–I is ternary plots of the proportion of the total number of dogs that are infected at equilibrium (prevalence). Again there is a shift from low density to high density with peak prevalence first corresponding to a large proportion of stray dogs, and then corresponding to a large proportion of owned free-roaming dogs. For all model outcomes, the general pattern is that if the proportion of confined dogs is fixed, then model outcomes are largely insensitive to whether the remaining dogs in the population are strays or owned free-roaming dogs.

### 3.2 Impact of Stray Dogs in the Presence of Vaccination of Owned Dogs

**Figure 6:**
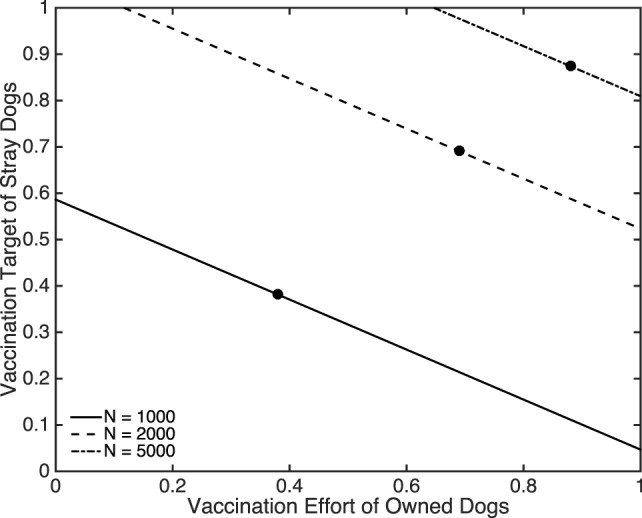
**The critical proportion of stray dog vaccination coverage required to prevent outbreaks as a function of the proportion of vaccinated owned dogs for three total population sizes comprising 50% stray, 25% owned free-roaming, and 25% owned confined**. Filled black circles denote when the vaccination efforts are equal for owned and stray dogs. For *N* = 1,000, (*a*_2_, *a*_3_, *γ*) = (0.1370, 0.0856, 3.6530 × 10^−6^). For *N* = 2,000, (*a*_2_, *a*_3_, *γ*) = (0.2740, 0.1712, 1.8265 × 10^−6^). For *N* = 5,000, (*a*_2_, *a*_3_, *γ*) = (0.6849, 0.4281, 7.3059 × 10^−7^). Other parameters are specified in Table 1.

We now suppose that a proportion of owned dogs (both free-roaming and confined) is vaccinated and ask what vaccination rate for strays is required to eliminate rabies. This vaccination target for strays, calculated as 1 − 1/*T*_1_, is shown in Figure 6 where we again show results for three scenarios: when the initial size of the dog population is 1,000, 2,000, and 5,000. For the two higher dog densities, the vaccination targets for the stray dogs are always greater than 50%, compared to much more manageable targets for the low density scenario. Figure 6 is a sharp reminder of the sensitivity of vaccination targets to dog density typically predicted when density-dependent transmission rates are assumed. The areas to the left of the lines indicate when rabies remains uncontrolled. For example, for *N* = 5,000 when 60% of owned dogs are vaccinated, rabies remains endemic even if all stray dogs were vaccinated. The points labeled A, B, and C in Figure 6 indicate where the critical vaccination effort for stray dogs is equal to the vaccination effort for owned dogs. The region to the left of these points represent when the critical vaccination effort required for stray dogs is higher than the level of vaccination achieved in the owned dogs and hence further increasing the effort directed toward stray dogs would be inefficient, misguided, and unrealistic (because vaccinating stray dogs is far more costly and difficult than vaccinating owned dogs).

**Figure 7:**
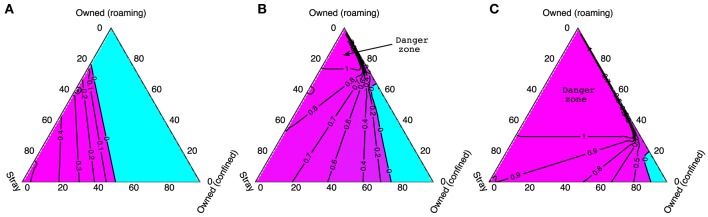
**The critical stray dog vaccination targets for different total dog population sizes: (A) 1,000, (B) 2,000, and (C) 5,000, when 70% of owned dogs are vaccinated**. Corners of the triangle represent when the dog populations contain only dogs of one type (as labeled); the edges when populations contain two dog types only, and the center represents when the population is made up equally of all three types. The blue area shows when a 70% vaccination rate of owned dogs alone is sufficient to bring *R*_0_ < 1. The region marked as “Danger zone” shows when vaccinating the entire stray dog population remains ineffective to control rabies despite a 70% owned dog vaccination rate.

In Figure 7, we again use ternary plots to show how stray vaccination targets depend on the composition of the dog population when 70% of the owned dog population is vaccinated. As was the case for Figures 5D–I, there is a shift between the low density case (*N* = 1,000) and the higher density cases (*N* = 2,000 and *N* = 5,000) where higher vaccination targets change from being associated with a high proportion of strays to a high proportion of owned free-roaming dogs. The light blue areas on these plots represent where vaccination of the stray dog population is not required as vaccinating 70% of the owned population is enough to eliminate rabies. The region where the stray vaccination target is larger than 1 (labeled danger zone) indicates when *R*_0_ > 1 despite a 70% owned dog vaccination rate (independent of the vaccination level of stray dogs). In this danger zone region, higher vaccination levels in the owned dog population are required before any effort directed toward the stray dogs could possibly be effective.

### 3.3 Effectiveness of Reducing the Stray Dog Population

**Figure 8:**
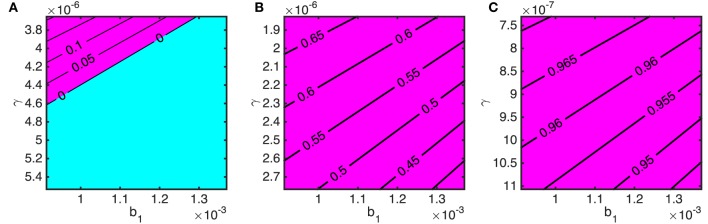
**The required stray dog vaccination targets to bring rabies under control in terms of natural death rate of stray dogs (*b*_1_) and strength of density-dependent mortality (*γ*) for varying stray dog population size (determined by *N*_1_ = (*a*_1_ − *b*_1_)/*γ*) when 70% of owned dogs are vaccinated**. Owned dog population sizes are *N*_2_ = *N*_3_ = **(A)** 250, **(B)** 500, and **(C)** 1,250. The blue area indicates when rabies is controlled without stray dog vaccination. The purple area shows the minimum proportion of stray dogs that must be vaccinated to bring *R*_0_ < 1.

We now consider the effects of varying the stray dog demographic parameters on the critical stray dog vaccination coverage; *b*_1_ represents the background mortality rate and *γ* determines the strength of density-dependence which sets the carrying capacity of the stray dog population. The three panels of Figures 8A–C correspond to having an owned dog population of 500, 1,000, and 2,500, respectively, and an owned dog vaccination rate of 70% is assumed.

The impact of a continuous stray dog culling program can, albeit simplistically, be modeled as an increase in *b*_1_ (death rate), and modifications to the environment that *reduce* the stray dog carrying capacity (such as improving disposal of food) as an *increase* in *γ*. The top left corners of the panels in Figure 8 correspond to *N*_1_ = 500 (Figure 8A), 1,000 (Figure 8B), and 2,500 (Figure 8C). The variation in the vertical axis (*γ*) spans a decreasing carrying capacity by 33% from top to bottom. Similarly, the variation from left to right in the horizontal axis (*b*_1_) is a 33% decrease in the average natural life span of a stray dog as representation of culling. For both parameters, increases result in reduced stray dog density but Figure 8 shows that shifts in *γ* are more effective at bringing down the required vaccination rates for stray dogs. Note that on the vertical axis, *γ* is plotted in descending values to reflect an increasing carrying capacity.

## 4 Discussion

We have used a differential equation model and the epidemiological quantity, *T*_1_, introduced by Roberts and Heesterbeek (19), to explore how vaccination targets for stray dogs might be expected to depend on dog population size and composition, stray dog demography, and on the vaccination rate that is achieved in the owned dog population. Our study shows that an increase in public knowledge around rabies (such as proper disposal of garbage) can be effective in reducing the required vaccination targets for stray dogs. We have found that the required stray dog vaccination rate is sensitive to the proportion of the total dog population that is owned and free-roaming. Indeed, a dog population consisting wholly of free-roaming owned dogs is predicted to require a higher vaccination coverage to ensure it is rabies-free, compared to any other mix of types, and even compared to a population consisting entirely of stray dogs.

This is not to say that populations of owned free-roaming dogs are more challenging for rabies control because owned dogs can be handled and are therefore easier to vaccinate. With this consideration, populations of stray dogs are clearly the most problematic. However, for example, our findings do underline concerns about rabies entering Australia’s northern communities where dog populations consist entirely of free-roaming dogs (30). There are typically no stray dogs in this population, but there are contacts with wild dogs (4). The insights we have presented here would predict that this mix of wild dogs and free-roaming domestic dogs could be highly vulnerable to a rabies incursion.

We emphasize that our modeling assumes that stray and owned free-roaming dogs have similar roaming behavior and hence similar exposure to infected dogs. The bottom right-hand corners of the ternary plots represent populations where a high proportion of the dog population is confined and these always correspond to the “safest” situations because the transmission rates of confined dogs are assumed to be much lower. The effect of higher proportions of stray dogs, which can be observed by traveling along the border of the ternary plot, is to subtly reduce *R*_0_ (Figures 5A–C). This is because higher demographic turnover means that an exposed stray dog is slightly less likely to survive long enough to become rabid.

In general, the three types would likely exist to some degree in all populations of dogs, though for specific regions and countries one or two of the types might be in negligible numbers. Defining these three types of dogs is a simplification of the varied and complex relationships between dogs and humans. Whether an owned dog is confined or allowed to roam, and even whether a dog has an owner or not, will have answers that vary from yes to no to everything in between. We therefore acknowledge that in reality the composition of a dog population and the validity of allocating every member to one of three types will vary from one region to another. Similarly, it is clear that dog densities vary from location to location, and contact rates too, so while we can endeavor to be in the right “ball park” it is not easy to be more precise than we have been unless we parameterize the model for a particular region where dog density, dog composition, and *R*_0_ are all known. Despite these caveats it is insightful to explore the consequences for rabies vaccination programs if there were groups of dogs that vary in their exposure (to bites from rabid dogs) as well as their freedom to infect other dogs should they become rabid.

The critical assumptions of this study are that dogs mix randomly at the population level, do not change from one type to another over their lifetime, and that every dog clearly belongs to one and only one type. The degree to which this is a simplification of reality, and what effect introducing further, more realistic, heterogeneity in the dog population would have on the epidemiological quantities that determine vaccination targets is unknown. Another core assumption is that the transmission rate between dogs has a linear relationship with density. This is a common feature of many rabies modeling studies (3, 23, 31); replacing the density-dependence with frequency-dependent contact in a standard SEIR compartmental model gives behavior that is discordant with observed dynamics (the dog population becomes extinct) (32), but there is conflicting evidence for density-dependent transmission and hence for the population thresholds one would expect to see as a result.

Our study highlights the importance of developing a better understanding of the dog ecology, such as dog population densities and degree of contact between the dogs, to guide future model development, improve model-based interpretation, and provide guidance to the design of vaccination programs. Our study provides a firmer scientific basis for vaccination programs aimed at stray or neighborhood dogs that cannot be handled and therefore cannot be brought to vaccination points. If the key dog ecology parameters can be estimated, then the theory provides definite vaccination targets, which can give greater confidence that a control program represents sufficient effort to eliminate canine rabies.

## Author Contributions

TL and SD together defined the research questions of the paper and designed the approach. TL performed much of the analytical work and obtained numerical solutions for the differential equations, created the figures, and helped write the manuscript. SD recovered many of the analytic results found by TL and led the writing of the manuscript.

## Conflict of Interest Statement

The authors declare that the research was conducted in the absence of any commercial or financial relationships that could be construed as a potential conflict of interest.
